# Evaluation of pediatric-specific resources to support utilization of the Wheelchair Skills Training Program by the users of the resources: a descriptive qualitative study

**DOI:** 10.1186/s12887-022-03539-0

**Published:** 2022-08-24

**Authors:** Béatrice Ouellet, Paula W. Rushton, Andrée-Anne Côté, Laurence Fortin-Haines, Emma Lafleur, Isabelle Paré, Melanie Barwick, R. Lee Kirby, Maxime T. Robert, François Routhier, Tatiana Dib, Yohali Burrola-Mendez, Krista L. Best

**Affiliations:** 1 Department of Rehabilitation, Université Laval, Quebec, QC Canada; 2 Center for Interdisciplinary Research in Rehabilitation and Social Integration, Quebec, QC Canada; 3 School of Rehabilitation, Université de Montréal, Montréal, QC Canada; 4 CHU Sainte-Justine Research Center, 5200, rue Bélanger Est, Montréal, QC H1T 1C9 Canada; 5 University of Toronto, Toronto, ON Canada; 6 SickKids Research Institute, Toronto, ON Canada; 7 Division of Physical Medicine and Rehabilitation, Dalhousie University, Halifax, NS Canada

**Keywords:** Occupational therapy, Wheelchair, Training, Knowledge transfer, Pediatric rehabilitation, Knowledge-to-action, Qualitative study

## Abstract

**Background:**

Children’s ability to engage in meaningful activities is positively influenced by their ability to move independently. Preliminary evidence in children suggests that wheelchair skills training improves wheelchair skills, which are important for independent mobility. The Wheelchair Skills Training Program is a standardized program to teach wheelchair skills. However, it is underutilized in pediatric rehabilitation settings. To increase its utilization, 3 pediatric-specific Wheelchair Skills Training Program resources related to indoor skills were developed (i.e., a storybook, four instructional posters, and a training workbook). This study aimed to describe occupational therapists’ (OTs) and pediatric manual wheelchair users’ (PMWUs) perceived satisfaction with the storybook, instructional posters and training workbook, and to explore their perceptions regarding the usability, relevance, and feasibility of these resources in pediatric rehabilitation settings.

**Methods:**

A descriptive qualitative design was used. Convenience samples of OTs and PMWUs were recruited in a rehabilitation center and affiliated schools. A focus group with OTs and semi-structured interviews with PMWUs were conducted by videoconference to obtain participants’ feedback on the resource prototypes and suggestions for improvement. Data were deductively analyzed using the Framework method.

**Results:**

Eight OTs and 5 PMWUs expressed general satisfaction with the resources, describing them as usable, relevant, and feasible to integrate into wheelchair skills training with novice wheelchair users and younger children. All OTs and 3 PMWUs expressed the desire to use the resources for wheelchair skills training. Two PMWUs perceived the resources were not relevant to them because they already mastered the skills. The participants suggested minor modifications for improving the resources (e.g., more action in the story, increased precision of illustrations related to the characters’ position in the wheelchair).

**Conclusion:**

OTs and PMWUs were satisfied with the resources, perceiving them to be applicable for training wheelchair skills among young children and novice wheelchair users. The resources represent a concrete solution to facilitate the use of the Wheelchair Skills Training Program in pediatric rehabilitation settings. Additional resources are needed to better reach older and more experienced PMWUs (i.e., of intermediate and advanced skill levels).

**Supplementary Information:**

The online version contains supplementary material available at 10.1186/s12887-022-03539-0.

## Background

Occupational therapists (OTs) are traditionally responsible for wheelchair skills training in Canada. One of the main goals for pediatric manual wheelchair users (PMWUs) is to promote their participation in activities that contribute to their development. Such activities include play, social interaction with peers, exploration opportunities, and independent mobility [1, 2]. Given that meaningful engagement in occupations is positively influenced by a child’s ability to move independently, training of wheelchair skills may facilitate participation, reduce dependency on parents for everyday mobility and prevent learned helplessness [1, 3].

The Wheelchair Skills Program (WSP) is a standardized program consisting of assessment (Wheelchair Skills Test) and training (Wheelchair Skills Training Program [WSTP]) protocols that OTs could use when working with PMWUs [4, 5]. The WSTP is effective, safe, and practical for improving wheelchair skills and confidence among adult users [6, 7]. The evidence has been replicated in various clinical populations, in low- and high-resourced settings, in a variety of clinical and community contexts, and using various training strategies [6, 7]. There is preliminary evidence in pediatrics that the WSTP improves wheelchair skills, wheelchair use confidence and participation in meaningful activities when offered individually, in groups or by a peer-trainer [5, 8, 9]. A qualitative survey [5] and a mixed-methods study, including Photovoice [9], revealed that parents perceived the WSTP to be beneficial for their children as it decreased shoulder pain and improved confidence, independence and safety when navigating environments.

Although the benefits of the WSTP have been documented, the quantity and quality of wheelchair skills training provided to children appear to be insufficient. Despite OTs perceiving wheelchair skills training to be important, a gap has been identified in the clinical uptake of effective programs [10, 11]. For example, a survey of 68 Canadian rehabilitation centers (43 of which offered services to children), revealed that most clinicians spent 2 h or less training manual wheelchair skills, and 18% provided no training at all [12]. Moreover, fewer than 30% of clinicians used an evidence-based training program, and the wheelchair skills training that was provided focused on basic skills [12]. Similar results were reported by OTs from a pediatric rehabilitation center and its affiliated schools in Montreal [10]. Given that many PMWUs have difficulties performing the skills necessary to participate in daily activities [2, 13], often rely on others for outdoor mobility [2] and are at risk of suffering wheelchair-related injuries (e.g., tips, falls, shoulder pain) [10, 14–16], it may be useful to develop additional resources to encourage the uptake of the WSTP and provision of training in pediatric rehabilitation settings.

Application of the Knowledge to Action (KTA) framework can optimize the translation of evidence into practice [17]; in this case, the use of the WSTP in pediatric rehabilitation settings. According to the KTA framework, knowledge translation is a dynamic process that includes a 3-phase knowledge creation funnel and 7-phase action cycle. Creation and action phases influence one another and can overlap [17]. Involving end-users (i.e., OTs and PMWUs) throughout the creation and action phases optimizes the chances that the knowledge will be tailored to their needs, thereby promoting the uptake in clinical practice. Adapting the knowledge to the local context is a critical action phase, which includes identifying required modifications to support pediatric considerations [17]. Daoust et al. [11] documented OTs’ concerns about using the WSP in a pediatric setting, namely improvements were needed to address playfulness of the resources, skill choice, practicality, pediatric specificity, and resource constraints. These findings identified a need to develop pediatric-friendly resources, which represents development of targeted knowledge translation tools and products in the knowledge creation funnel (i.e., third phase).

Three complementary resources (storybook, instructional posters, and training workbook) were developed by 14 members of our interdisciplinary research team (1 adult wheelchair user, 4 professional Masters of OT students, 4 researchers with expertise in wheelchair skills training, 1 researcher specialized in KT, 2 pediatric OTs, and 2 rehabilitation services managers). To produce a first version of the resources, a 3-step iterative process of creation (i.e., writing, drawing and layout by professional Masters of OT students), feedback (i.e., team meetings for sharing sample resources drafts and discussing improvements) and modification (i.e., tool refining) was conducted 3 times. According to the action cycle of the KTA framework, the next step was to assess barriers and facilitators to using these resources in the pediatric context among those positioned to provide and receive training [17, 18].

Primary users’ perceptions towards the components and characteristics of the KT resources are one of the most important facilitators and barriers to document (e.g., appreciation of the visual appearance, feedback on the understandability of the content for children, opinion on the usefulness of the resources to facilitate the training of wheelchair skills) [18]. The purpose of this study was to describe OTs’ and PMWUs’ perceived satisfaction with the storybook, instructional posters and training workbook, and to explore these users’ perceptions regarding the usability, relevance, and feasibility of these resources in pediatric rehabilitation settings.

## Methods

### Study design

A descriptive qualitative study was conducted using a focus group with OTs and semi-structured interviews with PMWCs following the COnsolidated criteria for REporting Qualitative research (COREQ) checklist [19]. The study was approved by the Sainte-Justine University Hospital Research Center Ethics Board. OTs and PWMUs’ parents provided informed consent. PMWUs provided assent prior the interview.

### Participants

Participants were recruited from the Marie Enfant Rehabilitation Center and its affiliated specialized elementary and high schools (i.e., Victor-Doré and Joseph-Charbonneau schools) using a convenience sampling method. These three settings offer rehabilitation to children and youths up to 21 years old with physical disabilities in Montreal, Canada. OTs were recruited between July and September 2020 using an information letter and a narrated PowerPoint presentation sent by email. OTs were eligible to participate if they: 1) were currently employed as an OT at the pediatric rehabilitation center or one of its affiliated schools; 2) provided wheelchair skills training services; and 3) were able to participate in an online focus group conducted in French. PMWUs were recruited between August and November 2020 using an invitation letter provided by their treating OT. PMWUs were eligible to participate if they: 1) were between 5 and 15 years old; 2) had received OT services at the pediatric rehabilitation center or one of its affiliated schools within the previous 3 months; 3) used a manual wheelchair at least 4 hours/day for at least the previous 6 months; 4) were able to understand simple instructions and participate in a French discussion online (i.e., by Zoom) for 60 minutes.

### Description of the new WSTP resources

The 3 resources addressed 4 indoor skills (of the 32 wheelchair skills included in the WSP manual version 5.1) including rolling forward a short distance, rolling backward a short distance, turning while moving forward, and picking objects from floor. These skills were selected as they are fundamental to learning more complex skills (e.g., ascending an incline, descending a curb) and are often necessary for PMWUs to navigate school and community settings (e.g., moving in narrow hallways, picking up a pen, and playing). The resources integrated some principles of motor learning (e.g., assisting with goal setting and motivation, and providing demonstration, extrinsic feedback, and verbal cues), were culturally and gender inclusive for use by diverse children (e.g., the characters had different skin colors and a gender-neutral physical appearance), and used child-friendly language. Similar characters and illustrations were used in the three complementary resources to ensure continuity, as each tool was created for a specific purpose in the training process but intended to be used as a package to facilitate the use of the WSTP for OTs and youths’ engagement.

#### Storybook

**Fig. 1:**
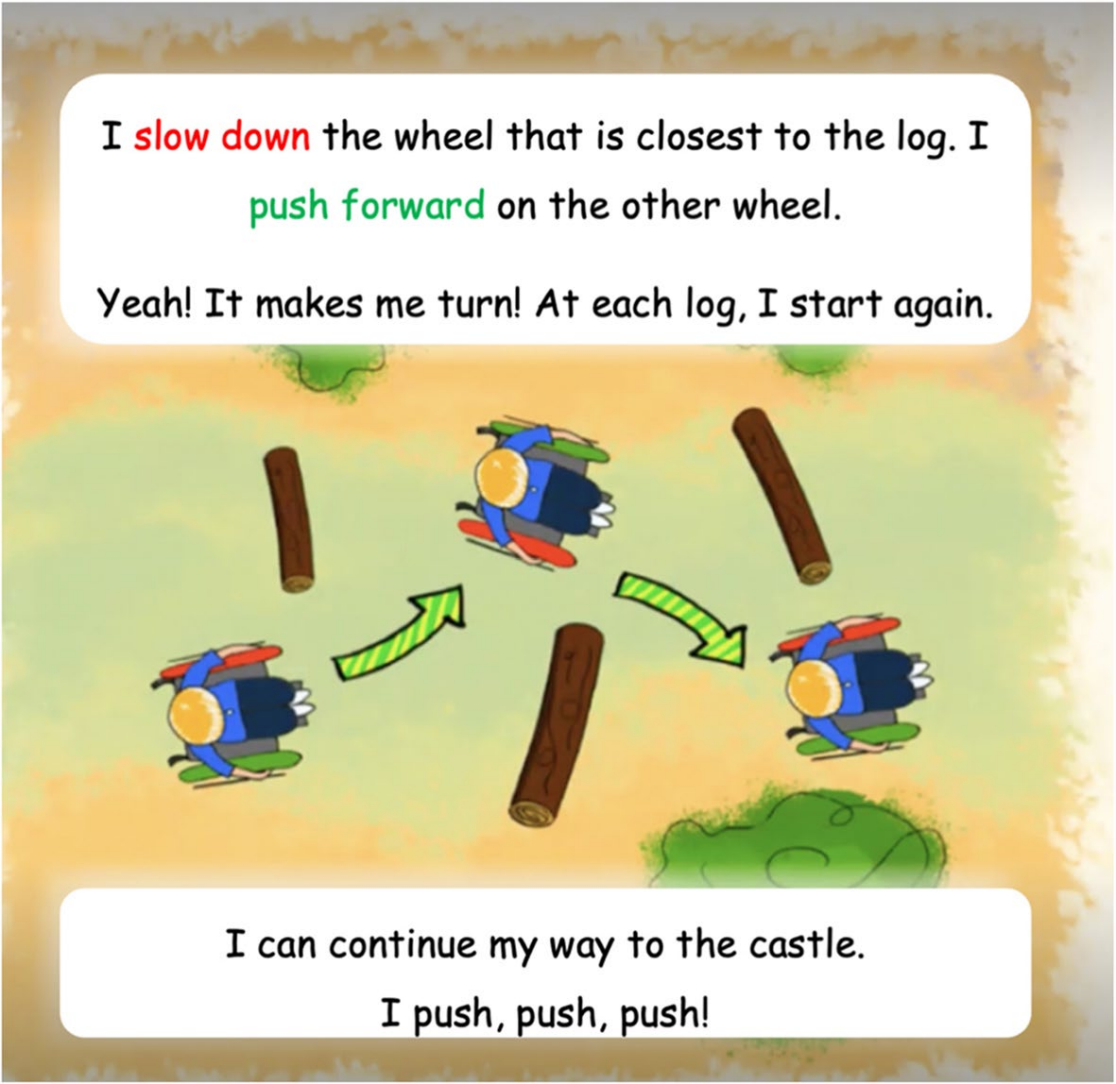
Storybook page on the skill “Turns while moving forward”

A 10-page storybook introduced wheelchair skills training to PMWUs in a stimulating and motivating way. The storybook was intended to create awareness (among PMWUs, their families, friends, and the multidisciplinary team) that PMWUs can learn wheelchair skills to facilitate independence and engagement in day-to-day activities. The storybook featured Gab (they/them), a manual-wheelchair-using school-aged child who goes on an adventure in the forest with their dog to discover a castle. The path to the castle is challenging and requires that Gab perform the 4 wheelchair skills. Gab describes the techniques used to perform the skills, and the images illustrate the movements. An example of a page of the storybook is presented in Fig. 1.

#### Instructional posters

**Fig. 2:**
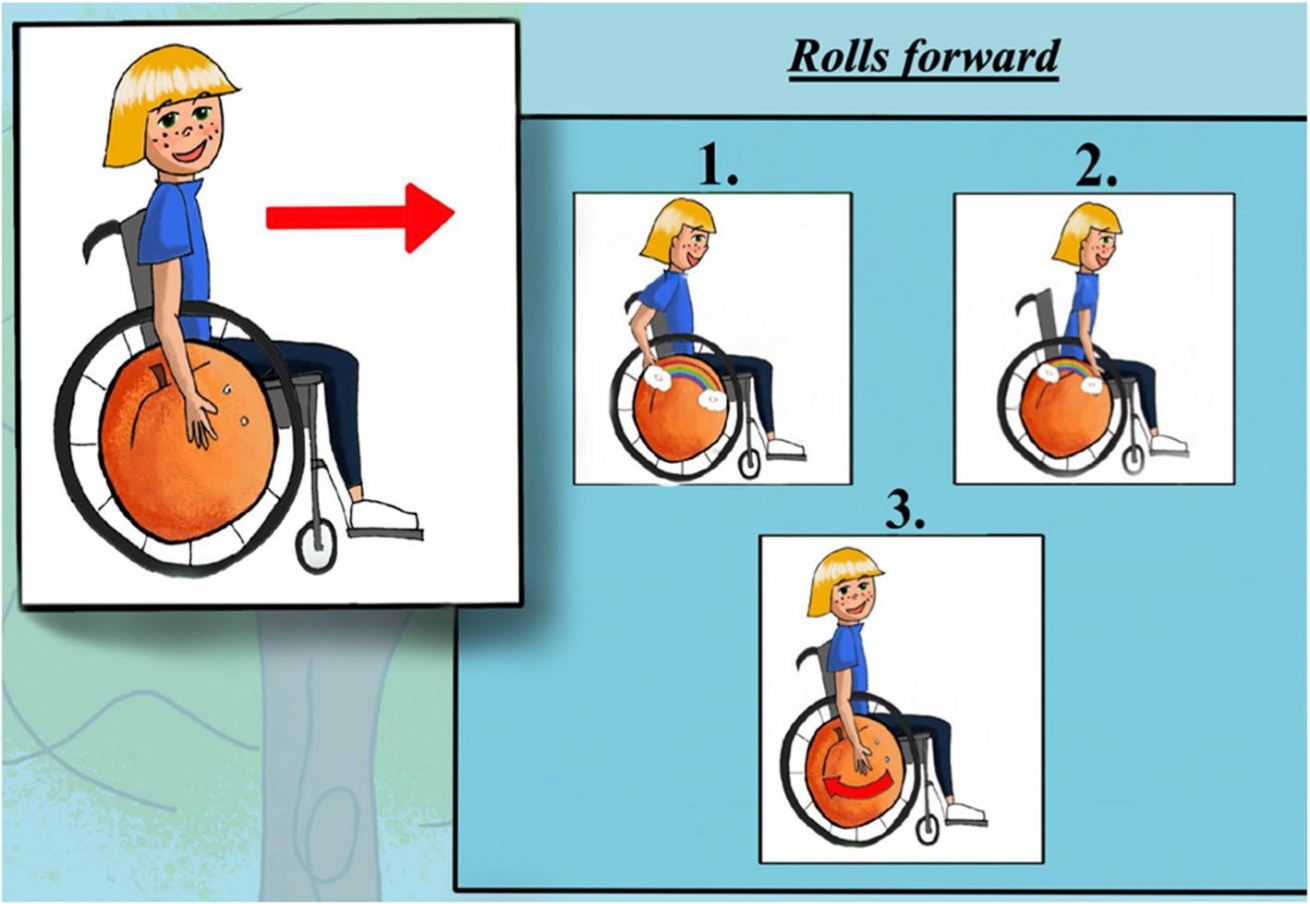
Poster on the skill “Rolls forward”

Four instructional posters (i.e., 1-page for each skill) could be used to provide visual support during training to help PMWUs understand the techniques required to perform the skills. They could also provide reminders in a variety of settings (e.g., home, school, rehabilitation center). Guided by principles of motor learning, the skills were divided into 3 smaller objective steps that were illustrated, with visual cues integrated within each image to facilitate learning and integration. An example of a poster is presented in Fig. 2.

#### Training workbook

**Fig. 3:**
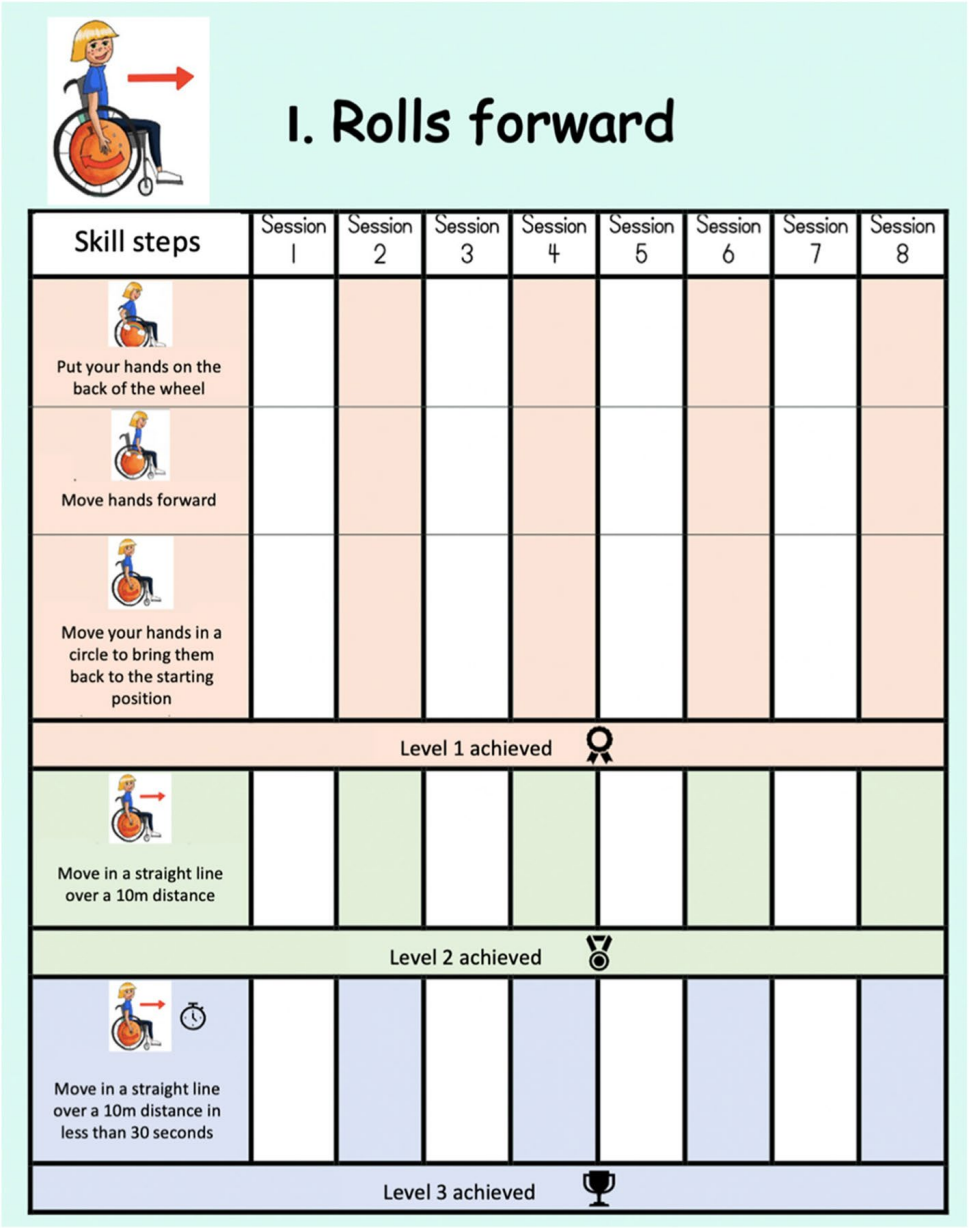
Training workbook page for the skill “Rolls forward”

The training workbook, intended to be used collaboratively by the OT, PMWU and parent, included three sections: 1) mobility goals (i.e., lines to write 5 specific, measurable, achievable, realistic and time-bound objectives [4]); 2) skill progression (i.e., 4 tables in which the child and the OT could draw smiley faces or add stickers when a skill step is accomplished); 3) comments (i.e., 4 pages with lines to write qualitative information on the skill performance). An example of a page of the training workbook is presented in Fig. 3.

### Procedures

Data collection began with a sociodemographic questionnaire for each sample: OTs (age, sex, work setting, years of experience, years working with PMWUs, frequency of wheelchair skills training and use of the WSTP using a 5-point Likert scale (i.e., never, rarely, sometimes, frequently, always) [12]; PMWUs (age, sex, years using a manual wheelchair, years using current wheelchair, previous wheelchair skills training, training location, and assistance needed for wheelchair mobility).

The WSTP resources were provided to participants in advance of the focus group or interviews to ensure familiarization to facilitate the discussions. The focus group and semi-structured interviews were conducted using online videoconferencing (Zoom) to respect the COVID-19 public health restrictions at the time of data collection. The semi-structured guides for the focus group and interviews were developed using the *Guide to Monitoring and Evaluating Knowledge Management in Global Health Programs’* indicators [20], which included: overall satisfaction (i.e., resources meet needs), usability (i.e., ease of use in terms of format [aesthetics], ease of understanding the content), and relevance (i.e., importance of content and applicability for OTs and PMWUs). The feasibility of implementing these 3 resources into clinical practice was also explored (i.e., capacity to use the resources regarding time, environment, and personal characteristics).

#### Focus group

One 90-minute focus group with OTs was cofacilitated by 4 OT student team members (AAC, IP, LFH, EL). The focus group started with a brief background on the WSTP, and the resources were presented to facilitate the discussion. Then, two 30-minute periods of discussion were conducted, separated by a 10-minute break, to respond to 8 open-ended questions and related probes. Examples of questions for each indicator included: “What is your first impression of these 3 resources?” (satisfaction), “What are your thoughts on the presentation and format of the resources?” (usability), “In an ideal context, how would you use the resources (OTs)?” (relevance), and “In your current clinical context, is it feasible to use these resources?” (feasibility). In addition, 3 quantitative, closed questions were asked at the end of the focus group using the Zoom poll feature that captures anonymous data. Specifically, for each tool, OTs were asked to rate on a 10-point scale (from 1 [not at all] to 10 [totally]), if the tool would help them to provide wheelchair skills training, and if the format and aesthetics would facilitate wheelchair skills training. They were also asked to respond to a dichotomous question (i.e., yes or no) reporting their intention to use the tool in their clinical setting. A PowerPoint presentation was used as a visual support to facilitate the discussion. To make the process more interactive and engaging, OTs were given access to a Google Slides document (i.e., separate shared PowerPoint) where they could write short answers to the questions at times specified by the facilitators. The focus group guide was piloted with 8 members of the research team (i.e., 1 adult manual wheelchair user, 2 OTs, 5 researchers), modifications were made according to their suggestions. The focus group was audio recorded.

#### Interviews

Sixty to 90-minute semi-structured interviews were conducted with PMWUs by an OT student team member (AAC). If PMWUs verbally expressed or physically demonstrated signs of fatigue or low concentration, the interviews were stopped for a short break or conducted over 2 sessions to ensure optimal participation. According to the participants’ preferences, a parent could accompany the PMWUs during the interview to help them with Zoom, rephrase questions or clarify answers. First, rapport building involved introductions and general questions about PMWUs’ interests. Then, PMWUs were asked 7 open-ended questions and probes for the storybook, 12 for the posters, and 14 for the training workbook. Examples of questions for each indicator included: “What do you like most and least about the tool?” (overall satisfaction), How do you like the characters? (usability), “Did the tool help you to learn new things for using your wheelchair?” (relevance). A PowerPoint presentation depicting the questions and resources was used as visual support for the interview. As well, 3 smiley-face pictograms (i.e., happy, neutral, sad) were used to facilitate children’s expression of their opinions regarding the resources. An OT student team member (AAC) pilot tested the interview with a PMWU to help ensure the questions were understandable for children and that they were able to express their points of view. The PMWU was an 8-year-old boy who had used a manual wheelchair for 6 years and his current wheelchair for 2 years. He had previously received manual wheelchair skills training in school. The semi-structured interview guide was modified according to his suggestions. The interviews were audio recorded.

### Data analyses

Descriptive statistics were calculated for the sociodemographic data (medians and interquartile ranges). The qualitative data were deductively analysed using the Framework method [21]. Focus group and interview recordings were transcribed verbatim and anonymized using pseudonyms. All focus group and interview transcriptions were entirely verified by a member of the team. Two matrices, one for the focus group (see Additional file 1) and one for the interviews (see Additional file 2), were collaboratively created by the research team and organized with the 4 indicators (overall satisfaction, usability, relevance, feasibility). The matrix for the interview was piloted and then refined with the interview of one of the PMWUs (participant’s pseudonym: James), as it was the one that covered the different themes more comprehensively. The focus group and 5 interview transcripts were independently coded by at least 2 team members. Although parents were not directly interviewed, the information provided when assisting their child was coded and considered in the interpretation of data to improve the understanding of PMWUs’ responses. Coding discrepancies were resolved by discussion. The research team met to interpret the data. Data saturation was not required to gather sufficient data to refine the resources in preparation for piloting. Neither the focus group and the semi-structured interview transcripts nor the analyses were returned for verification to the OTs and PMWUS (i.e., member checking) to reduce the study burden at a particularly uncertain and challenging time for health professionals and families (e.g., load shedding and homeschooling due to COVID-19). The direct quotes provided in this article have been translated from French to English by bilingual members of the research team. Quantitative focus group data were analyzed using descriptive statistics. Means were calculated, and data were visually illustrated in bar graphs with error bars to show the distribution of the data around the means.

## Results

Eight OTs participated in the focus group (five from Victor-Doré primary school, one from Joseph-Charbonneau high-school, and two from Marie Enfant Rehabilitation Center). Five PMWUs who received services from Marie Enfant Rehabilitation Center, or an affiliated school were interviewed. All PMWUs were accompanied by their mothers. Two mothers were not involved for the entire interview, remaining nearby to intervene in case their child needed help. Three mothers participated in the entire interview to rephrase questions and clarify their child’s answers. Tables 1 and 2 respectively present OTs’ and PMWUs’ demographics.

**Table 1:** Sociodemographic information of Occupational Therapists (*n* = 8)

Pseudonym	Age (years)	Sex	Experience at current site (years)	Experience with PMWUs (years)	Experience as an OT (years)	Frequency of wheelchair skills training	Frequency of WSTP use
	Median = 29		Median = 2.5	Median = 4.3	Median = 7.5		
	IQR = 17		IQR = 6.5	IQR = 8.8	IQR = 13.8		
Marie	44	Female	6	6	22	Often	Sometimes
Audrey	29	Female	2.5	2	6	Rarely	Sometimes
Alexandra	28	Female	2	2.5	4.5	Sometimes	Rarely
Sara	NP	Female	11	13	13	Rarely	Rarely
Nathalie	32	Female	2.5	8.5	9	Sometimes	Often
Anna	27	Female	2	2	3	Sometimes	Rarely
Kaitlyn	26	Female	2	2	3	Sometimes	Never
Julie	54	Female	29	29	30	Sometimes	Rarely

Legend *OT* Occupational therapist, *PMWUs* Pediatric manual wheelchair users, *WSTP* Wheelchair Skills Training Program, *SD* Standard deviation, *IQR* Interquartile range, *NP* Not provided, Frequency of wheelchair skills training and frequency of the WSTP use from the response choices: ‘never’, ‘rarely’, ‘sometimes’, ‘often’, ‘always’

**Table 2:** Sociodemographic information of PMWUs (*n* = 5)

Pseudonym	Age (years)	Sex	Experience using a manual WC (years)	Experience with current WC (years)	Previous training	Frequency of assistance	Type of assistance
	Median = 10		Median = 6	Median = 1			
	IQR = 4		IQR = 3	IQR = 1.5			
Lucia	6	Female	3	2	No	Always	S, VA, PAT, PAM
James	8	Male	5	0.5	No	Sometimes	PAT, PAM
Justine	10	Female	6	1	RC	Often	PAT, PAM-O
Thomas	12	Male	8	8	School	Sometimes	S, PAT, PAM-O
Catherine	12	Female	9.5	0.5	School	Sometimes	NP

Legend *WC* Wheelchair, *SD* Standard deviation, *IQR* Interquartile range, *RC* Rehabilitation center, Frequency of assistance from the response choices: ‘never’, ‘rarely’, ‘sometimes’, ‘often’, ‘always’, Type of assistance: *S *Surveillance, *VA *Verbal assistance, *PAT *Physical assistance for transfers, *PAM *Physical assistance for mobility, *PAM-O *Physical assistance for outdoor mobility only, *NP *Not provided

### Overall satisfaction

All OTs had positive first impressions of the 3 resources, 2 of whom (Anna and Sara) described them as “*turnkey*”, because they were ready to use in clinical settings to structure and deliver wheelchair skills training for PMWUs. OTs found the resources attractive, easy to use, playful, colourful, inclusive, and suitable for young children. OTs emphasized that the resources would meet their wheelchair skills training needs, as they may provide efficiencies in training, enhance children’s engagement and facilitate their knowledge. They also mentioned that the resources could be especially helpful for children with communication difficulties or intellectual disabilities. OTs felt the resources could assist with transitions to wheelchair use.

PMWUs had mixed first impressions of the resources. Three PMWUs appreciated the resources, as they learned new wheelchair skills or improved their techniques for performing them. However, 2 PMWUs (Justine and James) felt the resources were immature for their age and skills level. PMWUs found the storybook funny and clear. They particularly liked the dog, the castle, and the multi-colored socks. However, PMWUs expressed that the plot needed to be more exciting. For the posters, the “Rolls forward a short distance” skill was a favorite because of the rainbow used to illustrate how the arms should move in an arc pattern. In the training workbook, PMWUs appreciated that the table could be completed with their OT during training to track progress over time. They particularly liked the idea of using smiley faces or stickers to highlight their success.

### Usability

**Fig. 4:**
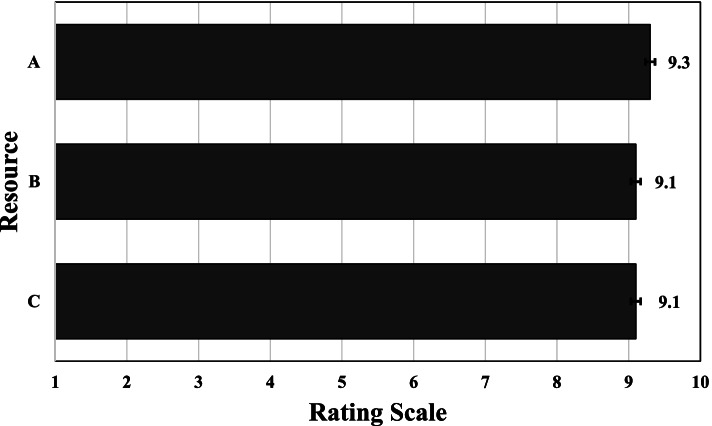
OTs’ perceptions: format and aesthetics of the resources could facilitate the training of wheelchair skills. Legend: Resource: Training book (**A**), Storybook (**B**), Posters (**C**). Question**:** On a scale of 1 to 10, do you think the format and aesthetics of the resource could make it easier to train wheelchair skills?

OTs considered that the format and aesthetics of all 3 resources could facilitate wheelchair skills training (see Fig. 4). Both OTs and PMWUs liked the colors, and OTs felt the use of primary colors provided good visual contrast. OTs appreciated the diversity of the characters (gender, nationality), suggesting that children connect more when resembling the characters. One PMWU (Justine) described the characters as “cool”, appreciating that the characters were in a wheelchair like her. In this regard, her mother emphasized: *“I think it is super that children can identify with someone in a wheelchair. That is something really good, that uh, uh can maybe a little repair children’s heart. Usually, children do not recognize themselves in stories.”* Regarding fonts, OTs felt the bold headings were easy to read, and they liked the selection of minimal yet effective words.

Some improvements were suggested to increase the usability of the tools. Two OTs (Sara and Audrey) suggested customizable versions of the tools (e.g., *“to have a version that we could modify, that we could personalize a bit to the needs of every child”*). OTs and PMWUs suggested that the illustrations of the wheelchair components should be more detailed, and body positioning on the wheelchair could be depicted more accurately. For example, a PMWU (James) stated “*The hand rims of the wheelchair are missing*. *Usually, you push on them to move forward*… *The fact that Gab puts his hands on his leg. I think it is not very clear. It looks like he’s trying to stop the wheelchair. His hand should be much closer to his thigh*”.

OTs appreciated the comprehensibility of the content, describing the language as suitable for young children and containing expressions frequently used in practice (e.g., “*push, push, push*”, “*yay*”). They explained that the presence of images, the bright and clear signs, and the use of keywords made it easier to understand for PMWUs, especially for those with language difficulties. Despite these elements, PMWUs needed support to use the resources. Two PMWUs needed help to read the resources, especially for the storybook. Four PMWUs expressed they needed explanations to understand the purpose of the posters and training workbook, and the contexts in which they could be used. Three PMWUs had difficulties understanding the “Objectives” and “Levels” sections of the training workbook, suggesting replacing the word “Objectives” with “Challenges”. One mother stated that these sections of the training workbook would be more relevant for OTs and parents than for children. One PWMU (Justine) said: “*Objectives make me uncomfortable. I don’t want to have mobility objectives. I see my wheelchair as an asset and I want it to be fun*”. PMWUs needed parental explanations, demonstrations, or manual guidance for all resources to understand the different steps to perform the skills and the tips and tricks (e.g., the rainbow visual cue to show the movement of the arms for the skills “Rolls forward a short distance” and “Rolls backward a short distance”). When understood, the tips and tricks were really appreciated by children: “*Now that I understand why there is a rainbow on the wheel, I really like it* [Justine]”. Both OTs and PMWUs expressed that using the same tips and tricks across resources can increase clarity and improve understanding. For example, for the skill “Rolls forward a short distance”, PWMUs noted that the visual cue is an apple in the storybook and a rainbow in the posters. For the skill “Turns while moving forward”, OTs expressed that the image depicted in top view in the storybook was easier to understand than in front view in the poster. In addition, a PMWU (Justine) expressed that a small description of each step required to perform a skill should be added on the posters.

OTs and PMWUs liked the presentation, sequencing, and progression to facilitate the acquisition of new wheelchair skills. When talking about the training workbook, a PMWU (James) indicated: “*the breakdown of each skill in small steps in the three levels gives children a lot of chances of success*”. However, OTs suggested a more in-depth breakdown of each skill, as PMWUs with learning difficulties may need some extra steps to reach a skill level. In this regard, OTs and PWMUs proposed to add levels (e.g., “*performed with supervision and assistance* [Kaitlyn]”) to be inclusive of children who cannot perform the skills independently. OTs and PMWUs proposed recommendations to facilitate the understanding of the skills steps (e.g., add a cloud and a sun on the rainbow to indicate where to put the hands on the rim at the beginning and at the end of the movement). They mentioned that the steps of the skill “Rolls backward a short distance” were presented in the wrong order on the poster and proposed: *“Look backward on the two sides should be the first step* (James, Catherine)*, because if you move backward, hit someone and then look backward, it’s not appropriate* (James)”.

### Relevance

**Fig. 5:**
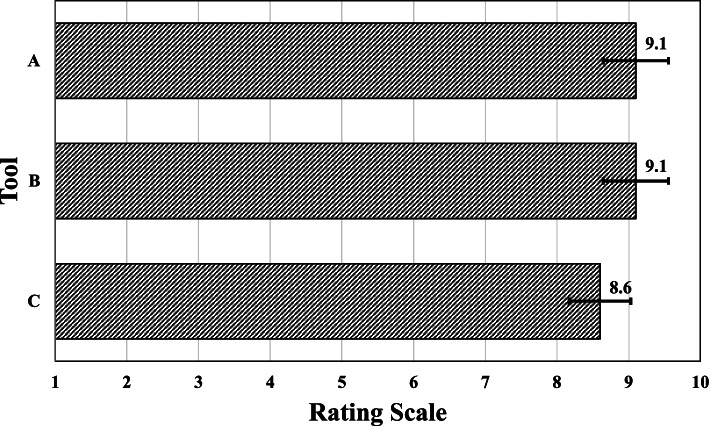
OTs’ perceptions on the resources’ relevance to help them in the training of wheelchair skills. Legend: Resource: Training book (**A**), Storybook (**B**), Posters (**C**). Question: On a scale of 1 to 10, do you think the resource will help you with manual wheelchair skills training?

OTs indicated that the resources would help them to provide wheelchair skills training to PMWUs (see Fig. 5). In fact, they expressed interest and willingness to implement them into practice immediately, *“as of tomorrow morning* (Marie, Sara, Anna, Audrey and Nathalie)*”*. The following quotes suggest intention and motivation for use: *“it is already much better than what I had done on my own, so it’s something very interesting with which I can start* (Marie)*”,* and *“I was missing a bit of motivation to start, and time also. But now, it makes me want to because I have something concrete. I can start from that* (Sara)*”*. OTs were motivated to use the resources, as they perceived them to be adapted for young children. For example, an OT (Audrey) from Victor-Doré elementary school expressed: “*They really target my clientele and are appropriate for my children’s age, especially because of the drawings that are for kids*”. OTs perceived possible time efficiencies and improved access to the WSTP, as the resources provide detailed information breaking the skills down for training in a pediatric-specific way. Furthermore, they expressed the resources could increase PMWUs’ engagement in the training process, as one OT (Kaitlyn) explained when talking about the training workbook: “*It’s fun, centred around them and personalized. They can take ownership of their objectives and progress. Usually, we guide them more, but with this tool, they are more involved in the training process*.*”*

Two PMWUs were motivated to practice wheelchair skills using the resources as they facilitated learning new skills through experience: e.g., “*Before I received the resources, I never tried to pick an object from the floor. Now, I try new things with my wheelchair* (Catherine)”. They also mentioned having learned more efficient techniques that could foster their mobility and independence: e.g., *“I learned that when I do big circles with my hands, like it is shown on the poster, I go faster. I can even move fast backward* (Lucia)*”*. One PMWU (James) expressed the desire to use the resources because they gave good reminders of the appropriate techniques he sometimes forgets: “S*ometimes you don’t realize it, but instead of stopping the wheel and turning, you go fast and turn. Then you may hit the wall”*. The resources motivated him to change these habits he had adopted over time and to practice improving his wheelchair skills. In contrast, 2 PMWUs (Justine and Thomas) did not perceive the benefits of modifying their techniques and were not interested in using the resources, even though one had neck and back pain while performing certain skills. Nonetheless, these PWMUs stated that the resources could be useful for beginner PMWUs.

OTs and PMWUs (and their mothers) perceived that the resources were less applicable to older PMWUs and experienced wheelchair users. The OT working at Joseph-Charbonneau High School felt the language, the presentation and the level of difficulty may be too young for her clients, except for PMWUs with intellectual disabilities. In this regard, when talking about the storybook, a 10-year-old PMWU (Justine) stated: “*It’s good, but it’s not appropriate for my age*” Another PMWU (James) mentioned: “*I have been using a wheelchair for a very long time, but, well, for sure it could teach them some things the children who use a wheelchair for a short time*”. Moreover, 2 mothers (of Justine and James) perceived that the skills addressed in the resources were too basic for their children who were “*More ready to learn how to go up sidewalk curbs, do wheelies, and go downstairs*”. For the storybook, PMWUs suggested that explaining why Gab goes to the castle and modifying the plot to include more action would better capture their attention and make it more interesting, particularly for older children. OTs and PMWUs suggested we develop other resources focused on higher-level skills and using a higher-level language to better engage experienced wheelchair users and older PMWUs.

OTs and mothers of PMWUs mentioned that other knowledge users could benefit from the resources, including but not limited to, other professionals involved in wheelchair provision (e.g., physiatrist, physiotherapists, social workers, psychologists), physical education teachers, family members, and peers. For example, the physical education teacher may benefit from using the posters, as *“She might be interested in having the posters to provide visual examples when practicing the skills with the children, to have them within her reach to support her teaching* (Marie)*”*. Moreover, the storybook could be used with non-PMWU peers to raise awareness about wheelchair use and capabilities of PMWUs: “*the storybook could be used at school to raise peers’ awareness, so that they can understand that the wheelchair is like the child’s legs* (Justine’s mother)”.

Most OTs envisioned how the 3 resources could be used at different points in the wheelchair provision process. OTs and PMWUs (and their mothers) suggested distributing the storybook before or when they first receive their wheelchair, as it could help families cope with the transition and enable children to identify with people living in similar situations. OTs also suggested the storybook could be used to introduce the training process: e.g., *“the story would be the introduction, the first thing to be presented to children and their family* (Kaitlyn)*”*. OTs proposed several contexts for use of the posters, including on the school walls, at Marie Enfant Rehabilitation Center and at home, as *“a reminder that these resources exist and of the techniques to perform the skills* (Kaitlyn)*”*, and to *“generalize the learning to different contexts* (Kaitlyn)*”*. OTs identified several applications for the training workbook, including as a *“communication tool* (Sara)*”*, to share information between OTs and parents or other team members about the skills trained, the techniques used, and children’s progress. OTs perceived the resources could be used for individual and group interventions. In addition, an OT (Anna) and a mother (Justine’s mother) mentioned that the resources could be relevant *“in many environments of a child’s life”* and *“at an early age”*, such as in kindergarten and at school.

### Feasibility

The OTs felt optimistic about having the time to use the resources in their daily practice, as they target the elements of the WSTP relevant to PMWUs. One OT (Anna) was confident they would be able to use the tool in the physical environment at Marie Enfant Rehabilitation Center: “*We have the space to provide training. We have long hallways and a laboratory at the Technopole*”. However, in an ideal world, OTs would have access to the same physical objects as presented in the resources to practice with PMWUs: “*I would have real wood logs...I would have the material to put the story in a tangible form* (Alexandra)”.

Three PMWUs mentioned that the method proposed for the skill “Picks objects from floor” was not feasible because their wheelchairs were too high. They suggested that alternative methods for performing this skill safely should be presented in the 3 resources (e.g., use long-handled reacher), which is also recommended in the WSTP [4]. A PMWU (Thomas) also had difficulties performing the skills using the method proposed in the resources due to difficulties with his left hand and back pain. These situations reinforced OTs’ suggestions of customizing resources for each child. Environmental barriers also limited PMWUs’ opportunities to practice the skills using the resources. For example, one PMWU (Catherine) was not able to use her training workbook at home for practicing the skills because she lived in a small apartment. Her mother also mentioned that the winter season was also a limiting factor.

## Discussion

In response to the need identified by Daoust et al. [11], condensed, ready-to-use, child-friendly resources were created to facilitate clinical use of the WSTP. Specifically, a storybook, 4 posters, and a training workbook were developed as a preliminary step to adapt the WSTP for the pediatric population. According to the KTA framework, these 3 resources are third-generation knowledge products with the potential to close the gap between the best available evidence on manual wheelchair skills training and clinical practices in pediatric rehabilitation settings [17, 18]. Given it is critical to consider context specific barriers and facilitators according to end-users [17, 18], OTs and PMWUs satisfaction and perspectives on the usability, relevance, and feasibility of the 3 resources were explored. While many aspects of the KT resources were perceived to be potentially useful to support wheelchair skills training in pediatric rehabilitation settings, refinements are needed to fully meet the needs of OTs and PMWUs. The Editorial Committee of the Wheelchair Skills Program Manual has formed a Pediatric Subcommittee to create special considerations for children. Recommendations from this study will be provided to the Subcommittee to support this initiative.

The inclusion of perspectives from OTs and PMWUs (and parents) on the 3 resources provided in-depth feedback from 2 primary end-users. Although fairly consistent, a few discrepancies were observed between OTs and PMWUs, reinforcing the critical importance of consulting both providers and users. Considering the perspectives of children and youth is a strategy to increase the chances that services align with their needs and preferences [22]. In this way, the current study supports with the United Convention on the Rights of the Child (1989) and the Nothing About Us Without Us movement (1998) by supporting the rights of children to express their points of views on the decisions pertaining to them. Research incorporating the perspectives of children with disabilities regarding the interventions they receive are limited [23, 24].

In general, OTs and PMWUs provided positive feedback regarding the use of the resources with elementary school children and novice wheelchair users. All OTs expressed the desire to use the resources with their clients as soon as possible, as they are easy to use in their context, may increase the efficiency of their interventions and make wheelchair skills training fun for children. Our findings suggest that the resources respond to the concerns regarding the playfulness of the WSTP, its practicality and its specificity in pediatrics, while decreasing the time barriers limiting its use (e.g., time to look at the WSTP protocol for planning the training sessions, to adapt the tips and tricks for children or to create learning material) [11, 12]. Three of 5 PMWUs perceived the resources could help them improve their mobility and were motivated to use them. The 2 PMWUs who felt they had already mastered the wheelchair skills (thus said they would not use the resources) were experienced wheelchair users who appeared to be ready to learn community and advanced skills. However, they did affirm that the resources could have benefits for younger children with less experience. Although this resonates with evidence supporting greater training effects in new wheelchair users [6], experienced children and adults have improved their wheelchair skills upon completion of the WSTP [5, 25]. Moreover, a PMWU (Thomas) had upper-body pain, a common wheelchair-related injury associated with overuse and poor propulsion techniques [15]. Pediatric-onset wheelchair use is associated with activity-limiting pain in adulthood [15]. Pain related to overuse might be prevented by using more efficient techniques to perform wheelchair skills [4, 15]. As children may tend to have difficulties imagining themselves in the future and understanding the relationship between the techniques they use and pain, it might be useful to raise their awareness on the long-term benefits of wheelchair skills training.

OTs, PMWUs, and mothers perceived that the resources could be used in a wide variety of contexts (e.g., home, kindergarten, school) and by diverse end-users (e.g., parents, peers, physical education teachers, physiatrist). This is particularly helpful given the need to increase children’s opportunities for wheelchair skills training and the importance of starting training as early as possible [5, 9, 26]. As health professionals working in rehabilitation and school settings have numerous competing priorities, training others such as parents, teachers or peers could alleviate some clinician burden [12, 27]. Wheelchair skills training could be delivered across the environments in which children live and grow (e.g., summer camps, sport activities, community organizations) to expose children to various contexts in which their manual wheelchair may be used. For example, power wheelchair mobility training was offered within a specialized summer camp to five school-aged children with severe cerebral palsy [28, 29]. After the camp, the impacts of the intervention extended beyond improving the children’s powered mobility skills to include positive changes in motor, cognitive, communication, and social skills [28, 29].

Despite the recognized benefits of improved independent mobility on global development among children with disabilities [30–32], parents are frequently reluctant to introduce wheelchair skills training early and commonly prioritize walking [11, 22, 33]. However, the literature does not indicate that wheelchair use limits the acquisition of walking skills [30]. Moreover, children with mobility impairments frequently mobilize using different strategies and equipment depending on the context. For example, children may walk or use a walker in their house and use a manual wheelchair for longer distances (e.g., going shopping with friends) [30]. In fact, the use of a wheelchair could limit fatigue and improve participation (e.g., by wheeling to the park and walking on the playground), thus reinforcing the importance of early familiarization with wheelchairs [30]. In this regard, participants suggested the storybook could be distributed to families during wheelchair prescription to enhance acceptance and awareness about the increased independence children could gain by moving independently around their environments. Given the lack of such resources to support children and families during wheelchair procurement, the storybook may help to ease the transition [22]. A precursor storybook may also help families to cope with the introduction to wheelchairs and could encourage wheelchair skills training. To date, such literature focuses mainly on power mobility [22]. More evidence is needed to understand how PMWUs and their families could use a precursor storybook to support skills building for manual wheelchairs.

The participants proposed several recommendations to improve the 3 WSTP resources. Adding more action to the plot, increasing playfulness, and writing 2 to 3 word-descriptions on the posters; all are relatively easy to modify. However, the suggestion to break the wheelchair skills into smaller steps (or add lower levels) in the training workbook to facilitate a level of success among all PMWUs may be harder to achieve. To date, there are no documented developmental milestones for manual wheelchair skills. Although the Wheelchair Skills Test classifies wheelchair skills in order of difficulty [4], a developmental sequence of wheelchair skills acquisition could be helpful for pediatric clinicians with concerns about skill choice [11]. A systematic review of pediatric occupational therapy interventions demonstrated that a common characteristic shared by the most effective interventions for improving motor skills follows a process of simplification and progression (i.e., breaking the skills down into smaller steps) [34]. Simplification and progression is one of the motor learning principles included in the WSTP [4], but more research is needed for pediatric wheelchair skills to provide a ‘just-right challenge’ and allow children to experience success throughout the learning process [11].

Other recommendations for improving the resources included improving the illustrations depicting how to perform the wheelchair skills, standardizing the visual cues in all 3 resources, and rendering the resources available in formats to allow OTs to customize them to the specific needs of their clients. Electronic fillable formats also facilitate communication and collaboration with parents, since OTs frequently use email to communicate with families of clients [11]. Posters for adults are already available in PDF on the WSP website [35] and the WSP Manual encourages clinicians to make adaptations to meet their clients’ needs [4, 11]. The WSP website appears to be a strategic platform to distribute the learning resources, as it has been accessed by 154,415 users from 196 countries; the WSP YouTube channel has had 179,324 views up to January 2022 [35].

### Limitations

Although our study was limited by a small sample size and the absence of data saturation, the triangulation of sources by the inclusion of OTs and PMWUs (and parents) allowed us to obtain in-depth feedback from different perspectives, and thus reinforce trustworthiness. Moreover, our sample of PMWUs had heterogeneous characteristics (i.e., younger and older, skills levels described ranged from novice to advanced, sometimes to always requiring assistance, previously having received training or not), which helped us to describe the opinion of a diversity of children. The transferability of the results to other pediatric contexts may be possible for the usability indicator, but limited for the satisfaction, relevance and feasibility indicators because site-specific characteristics could have influenced the perspectives. Despite the strategies used to elicit PMWU’s perceptions of the resources (e.g., PowerPoint visual support, pictograms, parental assistance), challenges arose during the interviews (e.g., brief responses, maintain attention and concentration, interruption by parents). The challenges may be explained by several factors. The interviews were conducted by a student who had limited experience in collecting qualitative data with children. Children may also have experienced fatigue during 60 and 90 minutes, or may have been distracted (e.g., by toys), potentially prompting parents to respond in their place. To prevent interference by parents, future studies could seek parent input on the resources before interviewing the PMWUs or provide ground rules regarding their participation. The resources were provided in advance to PMWUs to review the resources with their parents prior to the interview. Therefore, obtaining first impressions may have been limited. Finally, the 10-point scales for the 2 quantitative questions in the focus group ranged from 1 to 10. The fact that the lowest score is not 0 may have slightly increased the means and thus induced a small positive bias.

### Future research

Following the suggestions of OTs, PMWUs and parents, the resources developed in the current study should be refined and additional resources addressing skills at the community and advanced levels developed. Moreover, the resources should be translated in English to be accessible internationally. Components and characteristics of the resources are not the only types of barriers influencing their uptake in clinical practice. Cognitive, behavioral, and system process barriers (e.g., clinicians’ knowledge on manual wheelchair skills training, relative priority afforded to this intervention in pediatric rehabilitation settings), should be assessed to inform the selection of appropriate implementation strategies [17, 36]. In this regard, the resources should be pilot tested by OTs with PMWUs in the actual practice settings to better understand the barriers to uptake [17, 36]. In the long term, additional phases of the action cycle that follow the implementation of the resources (i.e., monitoring the use of the resources and evaluating their outcomes) should be carried out to promote their sustainable use [17].

## Conclusions

OTs and PMWUs (and parents) were generally satisfied with the new WSTP resources, perceiving them to be usable, relevant, and feasible for use in clinical practice. Most importantly, OTs felt inclined to use the resources within their practice. A storybook, posters and workbook may have the potential to support delivery of the WSTP in pediatric rehabilitation settings; however, this remains to be investigated. Additional resources that focus on intermediate and advanced skills are needed to cover the full range of WSTP skills and reach wheelchair users of different ages and skill levels.

## Supplementary Information

**Additional file 1.** Matrix for coding occupational therapists’ data following the Framework method [21].

**Additional file 2.** Matrix for coding pediatric manual wheelchair users’ data following the Framework method [21].

## Data Availability

The data generated and analysed in the study cannot be shared to respect participants’ confidentiality. The anonymous datasets forms are available from the corresponding author on reasonable request. The study materials to analyse the data are available in additional files.

## Abbreviations

OT(s): Occupational therapist(s)
PMWU(s): Pediatric manual wheelchair user(s)
WSP: Wheelchair Skills Program
WSTP: Wheelchair Skills Training Program
KTA: Knowledge to Action
KT: Knowledge transfer
