# Supplementary material for: Evaluation of pediatric-specific resources to support utilization of the Wheelchair Skills Training Program by the users of the resources: a descriptive qualitative study
Source: BMC Pediatr. 2022 Aug 24;22:500. doi: 10.1186/s12887-022-03539-0 (PMC9402274; doi:10.1186/s12887-022-03539-0)
Supplement: Supplementary file 1 — Additional file 1. Matrix for coding occupational therapists’ data following the Framework method [21]. [file 12887_2022_3539_MOESM1_ESM.docx]

**Additional File 1**

Matrix for coding occupational therapists’ data following the Framework method (Gale et al., 2013)

| **Global satisfaction:**  **Meets the intended user’s needs and expectations.** | | | | |
| --- | --- | --- | --- | --- |
|  | Storybook | Posters | Training workbook | In general |
| First impression |  |  |  |  |
| Time saving |  |  |  |  |
| User engagement |  |  |  |  |
| Playfulness |  |  |  |  |
| Other |  |  |  |  |
| **Usability:**  **Ease of targeted users to use the tools in terms of format and presentation (aesthetics)**  **Ease of targeted users to understand the tools’ content** | | | | |
|  | Storybook | Posters | Training workbook | In general |
| Colors |  |  |  |  |
| Characters |  |  |  |  |
| Fonts |  |  |  |  |
| Elements organization |  |  |  |  |
| Medium (paper/electronic) |  |  |  |  |
| Language level |  |  |  |  |
| Skills presentation |  |  |  |  |
| Other |  |  |  |  |
| **Relevance:**  **“Relevance” indicates that intended users find the information or knowledge applicable and important to their professional work.** | | | | |
|  | Storybook | Posters | Training workbook | In general |
| Intention to use |  |  |  |  |
| Collaborators |  |  |  |  |
| Context of use |  |  |  |  |
| Other |  |  |  |  |
| **Feasibility:**  **Capacity to use the tools regarding time, environment, and personal characteristics** | | | | |
|  | Storybook | Posters | Training workbook | In general |
| Available time in daily practice |  |  |  |  |
| Colleague openness |  |  |  |  |
| Opening of the organization - administration |  |  |  |  |
| Other |  |  |  |  |
| **Other comments** | | | | |
|  | Storybook | Posters | Training workbook | In general |
| Other |  |  |  |  |
| **Suggestions - Specific comments for changes** | | | | |
|  | Storybook | Posters | Training workbook | In general |
| Suggestions |  |  |  |  |
