# Supplementary material for: Evaluation of pediatric-specific resources to support utilization of the Wheelchair Skills Training Program by the users of the resources: a descriptive qualitative study
Source: BMC Pediatr. 2022 Aug 24;22:500. doi: 10.1186/s12887-022-03539-0 (PMC9402274; doi:10.1186/s12887-022-03539-0)
Supplement: Supplementary file 2 — Additional file 2. Matrix for coding pediatric manual wheelchair users’ data following the Framework method [21]. [file 12887_2022_3539_MOESM2_ESM.docx]

**Additional File 2**

Matrix for coding pediatric manual wheelchair users’ data following the Framework method (Gale et al., 2013)

| **Global satisfaction:**  **Meets the intended user’s needs and expectations.** | | | |
| --- | --- | --- | --- |
|  | Storybook | Posters | Training workbook |
| First impression | ☺ / 😐 / ☹ | ☺ / 😐 / ☹ | ☺ / 😐 / ☹ |
| Favorite elements |  |  |  |
| Disliked elements |  |  |  |
| Playfulness | ☺ / 😐 / ☹ | ☺ / 😐 / ☹ | ☺ / 😐 / ☹ |
| Other |  |  |  |
| **Usability:**  **Ease of targeted users to use the tools in terms of format and presentation (aesthetics)**  **Ease of targeted users to understand the tools’ content** | | | |
|  | Storybook | Posters | Training workbook |
| Tool’s visual appearance | ☺ / 😐 / ☹ | ☺ / 😐 / ☹ | ☺ / 😐 / ☹ |
| Characters | ☺ / 😐 / ☹ | ☺ / 😐 / ☹ | ☺ / 😐 / ☹ |
| Colors | ☺ / 😐 / ☹ | ☺ / 😐 / ☹ | ☺ / 😐 / ☹ |
| Structure or elements organization | ☺ / 😐 / ☹ | ☺ / 😐 / ☹ | ☺ / 😐 / ☹ |
| Understanding information | ☺ / 😐 / ☹ | ☺ / 😐 / ☹ | Objectives: ☺ / 😐 / ☹  Levels: ☺ / 😐 / ☹  Instructions: ☺ / 😐 / ☹  Comments: ☺ / 😐 / ☹  Other : |
| Skills presentation | Roll forward:  ☺ / 😐 / ☹  Pick objects from floor: ☺ / 😐 / ☹  Turn while moving forward: ☺ / 😐 / ☹  Roll backward:  ☺ / 😐 / ☹ | Roll forward:  ☺ / 😐 / ☹  Pick objects from floor: ☺ / 😐 / ☹  Turn while moving forward: ☺ / 😐 / ☹  Roll backward:  ☺ / 😐 / ☹ | Roll forward:  ☺ / 😐 / ☹  Pick objects from floor:  ☺ / 😐 / ☹  Turn while moving forward:  ☺ / 😐 / ☹  Roll backward:  ☺ / 😐 / ☹ |
| Other |  |  |  |
| **Relevance:**  **Indicates that intended users find the information or knowledge applicable and important.** | | | |
|  | Storybook | Posters | Training workbook |
| Tool catches children’s attention (interest them) | ☺ / 😐 / ☹ | ☺ / 😐 / ☹ | ☺ / 😐 / ☹ |
| Interest towards information on wheelchair skills | ☺ / 😐 / ☹ | ☺ / 😐 / ☹ | ☺ / 😐 / ☹ |
| Learn new things |  |  |  |
| Change the way a skill is performed | Roll forward:  ☺ / 😐 / ☹  Pick objects from floor:  ☺ / 😐 / ☹  Turn while moving forward:  ☺ / 😐 / ☹  Roll backward:  ☺ / 😐 / ☹ | Roll forward:  ☺ / 😐 / ☹  Pick objects from floor: ☺ / 😐 / ☹  Turn while moving forward:  ☺ / 😐 / ☹  Roll backward:  ☺ / 😐 / ☹ | Roll forward:  ☺ / 😐 / ☹  Pick objects from floor:  ☺ / 😐 / ☹  Turn while moving forward:  ☺ / 😐 / ☹  Roll backward:  ☺ / 😐 / ☹ |
| Encourage practice | ☺ / 😐 / ☹ | ☺ / 😐 / ☹ | ☺ / 😐 / ☹ |
| Desire to use the tool | ☺ / 😐 / ☹ | ☺ / 😐 / ☹ | ☺ / 😐 / ☹ |
| Other |  |  |  |
| **Feasibility:**  **Capacity to use the tools regarding time, environment, and personal characteristics** | | | |
|  | Storybook | Posters | Training workbook |
| Feasibility to perform the skills with the wheelchair and in the environment |  |  |  |
| Other |  |  |  |
| **Other comments** | | | |
|  | Storybook | Posters | Training workbook |
| Other - children |  |  |  |
| Other - parents |  |  |  |
| **Suggestions - Specific comments for changes** | | | |
|  | Storybook | Posters | Training workbook |
| Suggestions - children |  |  |  |
| Suggestions - parents |  |  |  |
